# Ceftriaxone attenuates Poly I:C–induced neuroinflammation *in vitro* by modulating glutamate transport, synaptic integrity, and immunometabolic reprogramming

**DOI:** 10.3389/fncel.2025.1684398

**Published:** 2025-10-28

**Authors:** Xizi Shi, Yesheng Sun, Shirin Hosseini, Fangfang Chen, Thekla Cordes, Kristin Michaelsen-Preusse, Martin Korte

**Affiliations:** 1 Department of Cellular Neurobiology, Zoological Institute, TU Braunschweig, Braunschweig, Germany; 2 Neuroinflammation and Neurodegeneration Group, Helmholtz Centre for Infection Research, Braunschweig, Germany; 3 Department of Bioinformatics and Biochemistry, Braunschweig Integrated Centre of Systems Biology (BRICS), Technische Universität Braunschweig, Braunschweig, Germany; 4 Research Group Cellular Metabolism in Infection, Helmholtz Centre for Infection Research, Braunschweig, Germany

**Keywords:** neuroinflammation, Poly I:C, ceftriaxone, LTP, GWAS

## Abstract

**Introduction:**

Neuroinflammation triggered by viral infections is increasingly recognized as a driving force in neurodegenerative disease, promoting chronic neuronal injury and cognitive decline. A central mechanism in this process is impaired glutamate clearance due to downregulation of the astrocytic glutamate transporter GLT-1 (EAAT2/SLC1A2), which exacerbates excitotoxicity and neuronal death.

**Methods:**

In this study, we assessed the neuroprotective effects of the β-lactam antibiotic ceftriaxone—a known upregulator of GLT-1—in an *in vitro* tri-culture model of neurons, microglia, and astrocytes challenged with the viral mimic polyinosinic:polycytidylic acid (Poly I:C).

**Results and discussion:**

Poly I:C exposure elicited robust microglial and astrocytic activation and increased levels of TNF-α, IL-6, and IL-10. Concomitantly, we observed significant downregulation of GLT-1, synapse loss, impaired synaptic plasticity, and disrupted amino acid metabolism. A complementary Mendelian randomization analysis of GWAS data revealed that genetically determined alterations in plasma amino acid levels are significantly associated with the risk of five major neurodegenerative disorders, underscoring the role of metabolic dysregulation in disease pathogenesis. Treatment with ceftriaxone effectively reversed the Poly I:C–induced phenotypes: GLT-1 expression, dendritic spine density, and measures of synaptic plasticity were all restored, and abnormalities in amino acid and tricarboxylic acid cycle metabolites normalized. These findings highlight ceftriaxone’s multifaceted neuroprotective profile—modulating glutamate homeostasis, preserving synaptic integrity, and rebalancing metabolic pathways—and support its potential as a therapeutic agent to prevent neuronal degeneration in the context of virus-driven neuroinflammation.

## Introduction

Neuroinflammation triggered by viral infections is a pivotal factor in the progression of neurodegenerative diseases, leading to sustained neuronal damage and cognitive decline (Przedborski et al., 2003). Infections by viral or bacterial pathogens reactivate CNS-resident immune cells, including microglia and astrocytes, resulting in the release of pro-inflammatory cytokines such as tumor necrosis factor-alpha (TNF-α) and interleukin (IL)-6 (Hickman et al., 2018; Giovannoni and Quintana, 2020; Zhang et al., 2023). This inflammatory response not only induces neuronal death but also perpetuates a chronic inflammatory cycle through the release of damage-associated molecular patterns (DAMPs) by dying neurons, further reactivating immune cells (Lotz et al., 2021). Genome-wide association studies (GWAS) have increasingly linked viral infections to an elevated risk of various neurodegenerative diseases, underscoring the significance of neuroinflammation in disease pathogenesis (Levine et al., 2023).

A critical component in this process is the dysfunction of Glutamate Transporter 1 (GLT-1, EAAT2/SLC1A2), predominantly expressed by astrocytes. GLT-1 plays a crucial role in maintaining synaptic integrity by rapidly removing glutamate from the synaptic cleft, thereby preventing excitotoxicity—a pathological condition caused by excessive glutamate accumulation that can lead to neuronal damage and death (Andersen et al., 2021). The glutamate-glutamine cycle, which involves both presynaptic glutamatergic neurons and astrocytes, is intimately connected to neuronal and astrocytic energy metabolism via the tricarboxylic acid (TCA) cycle. Disruption of GLT-1 impairs glutamate clearance, leading to altered amino acid flux and metabolic imbalances that contribute to synaptic and neuronal dysfunction (Estrada-Sanchez et al., 2009; Faideau et al., 2010; Woltjer et al., 2010; Scott et al., 2011).

In neurodegenerative diseases such as Alzheimer’s disease (AD), GLT-1 levels are significantly reduced. This decrease contributes to increased glutamate levels and excitotoxicity, which are associated with cognitive dysfunction and the progression of AD (Hoshi et al., 2018). GLT-1 expression is also significantly reduced in various infections, including Toxoplasma gondii (David et al., 2016) and human coronavirus (Brison et al., 2011). This reduction impairs glutamate uptake by astrocytes, disrupting glutamate homeostasis and potentially leading to neuronal dysfunction following infection.

Enhancing GLT-1 expression and functionality presents a promising therapeutic strategy to counteract neuroinflammation following viral infection and subsequent neurodegeneration. Ceftriaxone, a β-lactam antibiotic, has emerged as a potent enhancer of GLT-1 expression (Rothstein et al., 2005; Tai et al., 2019). Previous studies have demonstrated that ceftriaxone effectively restores GLT-1 levels, thereby mitigating glutamate-induced excitotoxicity and providing neuroprotection in various models of neurodegeneration (Rothstein et al., 2005; Tai et al., 2019). However, the precise role of GLT-1 in the early stages of neuroinflammation, particularly within viral infection models, remains inadequately understood.

In light of the limited availability of effective antiviral drugs for CNS infections and the scarcity of targeted treatments for neurodegenerative diseases, this study aimed to investigate the potential of the antibiotic ceftriaxone to manipulate GLT-1 expression and thus prevent neurodegeneration following viral infection. We selected ceftriaxone because it robustly upregulates GLT-1 and enhances glutamate clearance in the CNS, providing a mechanistically targeted rescue consistent with prior work (Rothstein et al., 2005). Ceftriaxone also has a substantial translational safety/pharmacokinetic record from ALS trials (despite lack of efficacy), which strengthens its relevance as a tool compound in our *in vitro* paradigm (Cudkowicz et al., 2014). Notably, reports of ceftriaxone-associated neurotoxicity are rare and typically reversible (e.g., encephalopathy, seizures), and there is no evidence for progressive neurodegeneration linked to its clinical use (Grill and Maganti, 2011).

Poly I:C–induced neuroinflammation in our neuron–microglia–astrocyte co-culture triggered robust glial activation, elevated TNF-α and IL-6, and downregulated the astrocytic glutamate transporter GLT-1. This cascade disrupted amino-acid metabolism, reduced dendritic spine density, and impaired long-term potentiation. Remarkably, ceftriaxone co-treatment reversed glial hypertrophy, restored GLT-1 expression, normalized metabolite profiles, and rescued both synaptic structure and function. Complementary Mendelian randomization of GWAS data further linked nine plasma amino acids to the risk of five neurodegenerative diseases, underscoring the importance of metabolic homeostasis. Together, these results position ceftriaxone as a promising approach to preserve glutamate clearance, curb inflammation, and protect neurons in virus-triggered neurodegenerative scenarios.

## Results

To model neuroinflammation, we established a triple co-culture of primary embryonic hippocampal neurons and astrocytes, maintained for 21 days *in vitro*, into which we then introduced microglia derived from pure microglia cultures. After allowing 48 h for microglial integration, we mimicked viral-induced inflammation by treating the cultures with polyinosinic:polycytidylic acid (Poly I:C, 25 or 50 μg/mL)—a synthetic analog of double-stranded RNA that elicits innate immune responses akin to those seen in viral infections. To assess ceftriaxone’s neuroprotective effects, we applied 100 μM ceftriaxone either alone or concurrently with Poly I:C for 24 or 48 h. This setup enabled us to examine how ceftriaxone modulates Poly I:C–driven inflammatory processes within an integrated neuron–astrocyte–microglia network.

### Poly I:C elevates cytokine production and induces microglial hypertrophy: ceftriaxone reverses hypertrophy without modulating cytokine levels

To elucidate the inflammatory response triggered by the administration of Poly I:C and to explore the potential beneficial effects of ceftriaxone, microglial reactivity and cytokine production were investigated.

**Figure 1:**
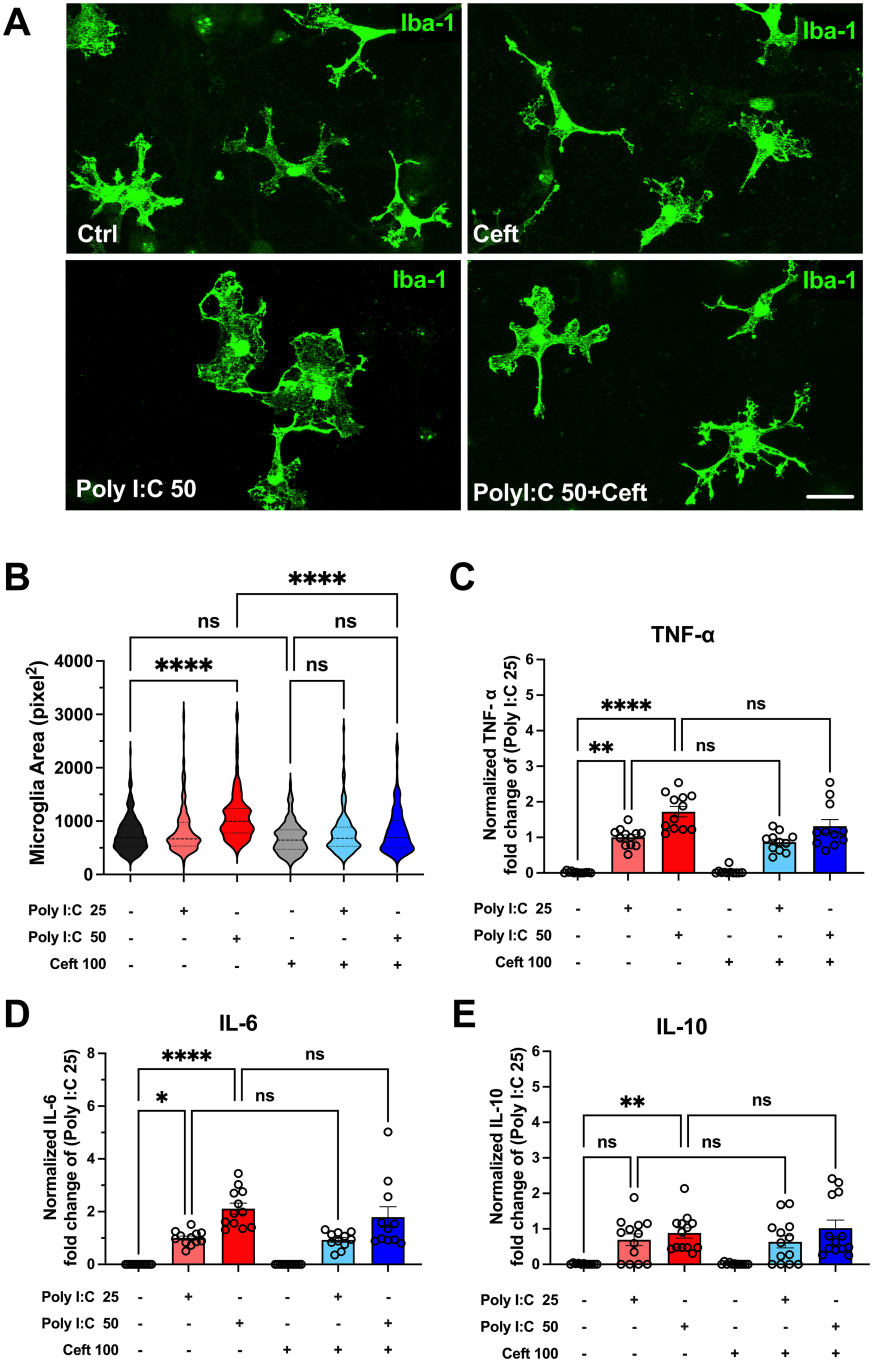
Impact of Poly I:C treatment and ceftriaxone co-treatment on cytokine levels and microglial morphology after 24 h. Poly I:C administration induced microglial hypertrophy and exhibited a dose-dependent increase in cytokine levels (TNF-α, IL-6, IL-10). Although ceftriaxone treatment did not inhibit cytokine secretion, it effectively reversed the Poly I:C-induced morphological changes in microglia. **(A)** Representative images of microglia across different treatment groups. Microglial cells were stained with an Anti-IBA1 antibody (green); **(B)** Quantitative analysis of microglial area. After 24 h of treatment with Poly I:C 50 μg/mL, microglia exhibited a significant increase in cell area, indicative of activation. In contrast, the Poly I:C 25 μg/mL group showed no significant change. Co-treatment with ceftriaxone effectively mitigated the Poly I:C-induced enlargement of microglia. Data are presented as mean ± SEM (*N* = 1,075, derived from 3 to 4 different batch experiments); **(C–E)** Levels of TNF-α, IL-6 and IL-10 measured by ELISA, normalized to the respective Poly I:C 25 μg/mL group. Poly I:C treatment resulted in a significant, dose-dependent increase in all three cytokines. Co-treatment with ceftriaxone did not significantly alter cytokine levels compared to Poly I:C treatment alone. Data are presented as mean ± SEM (*p < 0.05, **p < 0.01, ***p < 0.001, ****p < 0.0001; ns, not significant); scale bar 10 μm.

As a first step, to determine whether Poly I:C stimulation leads to microglial activation, IBA1 immunolabeling was employed for morphological analysis (Figure 1A). IBA1 is a reliable marker of microglial activation, playing a crucial role in the complex landscape of neuroinflammation. It facilitates the assessment of the morphological changes that arise when microglial cells interact with their environment (Guo et al., 2022).

After 24 h of treatment, microglia exposed to 50 μg/mL Poly I:C exhibited a significant increase in cell area, indicative of activation (Figure 1B). In contrast, the 25 μg/mL Poly I:C group showed no substantial change in microglial area. Notably, co-treatment with ceftriaxone effectively prevented the Poly I:C-induced enlargement of microglia, suggesting that ceftriaxone can partially mitigate microglial activation triggered by Poly I:C.

Cytokines such as TNF-α, IL-6, and IL-10 are pivotal mediators of the immune response, providing insights into the extent and nature of inflammation. Therefore, cytokine concentrations in the culture supernatant were measured using an ELISA assay to determine the inflammatory response following Poly I:C treatment and the role of ceftriaxone. After 24 h, Poly I:C administration resulted in a significant, dose-dependent increase in the levels of TNF-α, IL-6, and IL-10 (Figures 1C–E). Specifically, higher doses of Poly I:C corresponded to greater cytokine secretion. Interestingly, cultures co-treated with ceftriaxone and Poly I:C maintained cytokine levels comparable to those treated with Poly I:C alone, indicating that ceftriaxone does not inhibit cytokine release induced by Poly I:C. Conversely, treatment with ceftriaxone alone did not elevate TNF-α, IL-6, or IL-10 levels, suggesting its safety in the absence of an inflammatory stimulus.

### Astrocyte activation, glutamate transporter regulation, and mitochondrial integrity: protective effects of ceftriaxone

**Figure 2:**
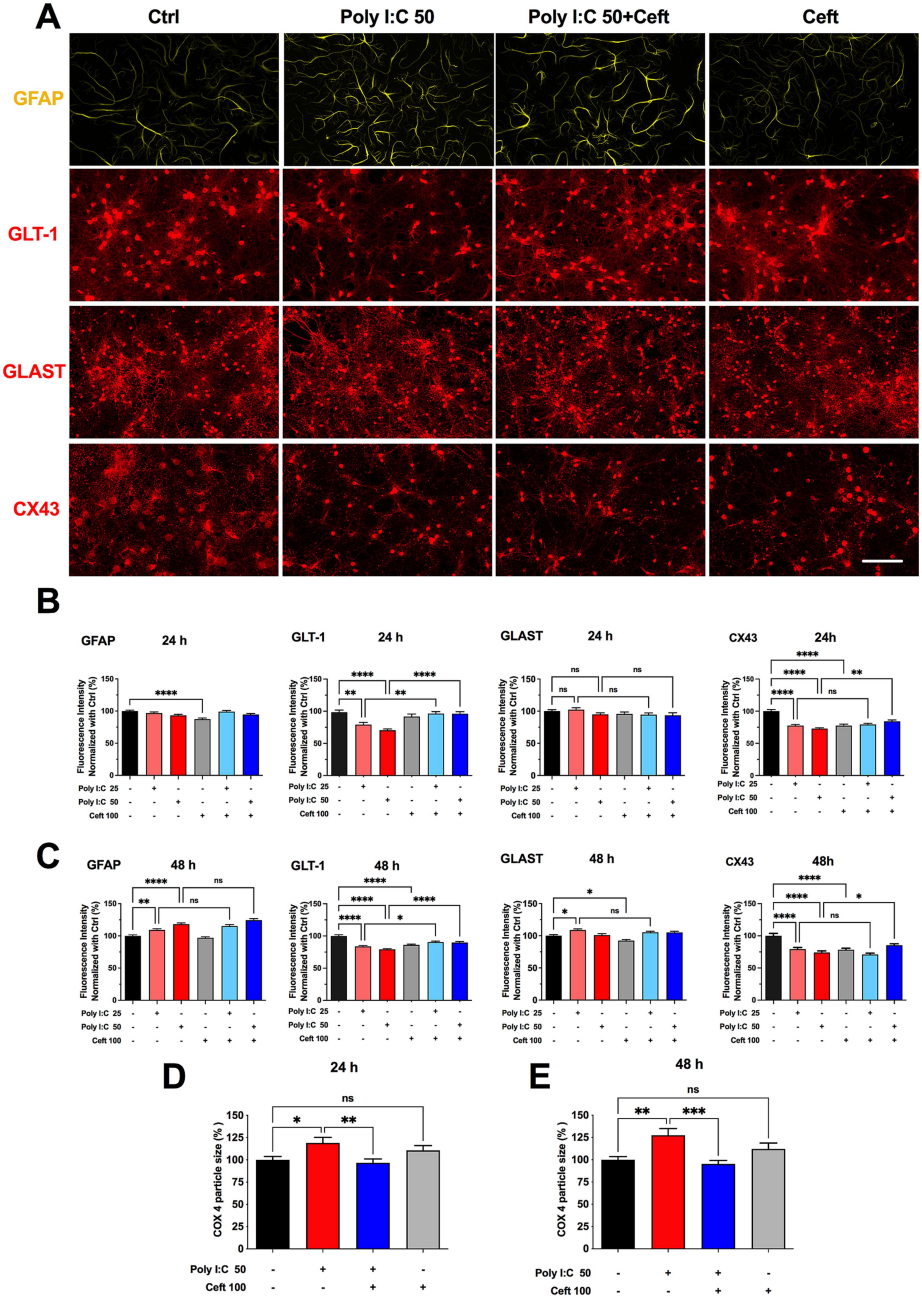
Effects of Poly I:C and ceftriaxone on astrocytic GFAP, GLT-1, GLAST, and CX43 expression at 24 and 48 h. Poly I:C treatment modulated the expression of astrocytic markers GFAP, GLT-1, GLAST, and CX43 in a time- and dose-dependent manner. While ceftriaxone effectively restored GLT-1 expression to control levels, it did not significantly alter the expression changes of GFAP, GLAST, or CX43 induced by Poly I:C. **(A)** Representative images of immunocytochemistry of astrocytes stained with anti-GFAP (green) and co-immunostained for GLT-1 (red), GLAST (red), or CX43 (red), respectively, across different treatment groups. Scale bar: 100 μm. **(B,C)** Quantified and normalized expression levels of GFAP, GLT-1, GLAST, and CX43 at 24 h **(B)** and 48 h **(C)** post-treatment. 24 h: GFAP (*N* = 360), GLT-1 (*N* = 205), GLAST (*N* = 237), CX43 (*N* = 359); 48 h: GFAP (*N* = 360), GLT-1 (*N* = 360), GLAST (*N* = 360), CX43 (*N* = 358); **(D,E)** Comparison of normalized COX4 particle size at 24 h and 48 h of the different groups. 24 h: *N* = 233; 48 h: *N* = 221. Data are presented as mean ± SEM; Statistical significance is indicated as follows: *p < 0.05, **p < 0.01, ****p < 0.0001; ns, not significant.

Astrocytic responses to Poly I:C were assessed by immunocytochemistry, with GFAP as a marker of astrogliosis. We also visualized the two principal astrocytic glutamate transporters -GLT-1, which mediates roughly 90% of extracellular glutamate uptake (Tanaka et al., 1997), and GLAST, enriched in perisynaptic processes and astrocyte somata (Achicallende et al., 2022). Because gap-junction coupling influences GLT-1 levels (Figiel et al., 2007), we included staining for connexin 43 (Cx43). Representative immunolabeling of GLT-1, GLAST, and Cx43 is shown in Figure 2A.

After 24 h, neither 25 nor 50 μg/mL Poly I:C altered GFAP levels, whereas ceftriaxone alone significantly reduced GFAP expression. Co-treatment with Poly I:C and ceftriaxone maintained GFAP at control levels. At 48 h, Poly I:C induced a dose-dependent increase in GFAP, and ceftriaxone co-administration did not prevent this upregulation. Ceftriaxone alone remained indistinguishable from control at 48 h (Figures 2B,C).

Poly I:C elicited a dose-dependent downregulation of GLT-1 after 24 h, an effect completely prevented by ceftriaxone co-treatment, which restored GLT-1 to control levels (Figure 2B). This protective effect persisted at 48 h (Figure 2C). In contrast, GLAST remained unchanged after 24 h of Poly I:C but was upregulated in the 25 μg/mL group at 48 h; the 50 μg/mL group showed no change. Notably, ceftriaxone alone reduced GLAST expression at 48 h (Figure 2C). Connexin 43 (Cx43) levels fell in response to both Poly I:C doses and to ceftriaxone alone. While ceftriaxone failed to reverse the Cx43 decrease at 25 μg/mL Poly I:C, it partially rescued Cx43 expression in cultures treated with 50 μg/mL Poly I:C (Figures 2B,C).

Astrocytes depend on healthy mitochondria to meet their high metabolic and signaling demands, yet many proinflammatory stimuli that drive astrocyte activation also disrupt mitochondrial function (Gollihue and Norris, 2020). To probe astrocytic bioenergetics, we focused on COX4, a core subunit of cytochrome c oxidase (complex IV)—the terminal enzyme in the electron transport chain that powers oxidative phosphorylation (Hartley et al., 2019). By combining anti–COX4 and anti-GFAP immunocytochemistry, we could specifically visualize and quantify mitochondrial network changes within GFAP-positive astrocytes. Particle analysis of COX4 staining revealed that Poly I:C treatment significantly enlarged mitochondrial puncta after 24 h—suggesting swelling or early network remodeling—an effect fully normalized by ceftriaxone. These alterations became even more pronounced at 48 h (Figures 2D,E), underscoring the vulnerability of astrocytic mitochondria to neuroinflammatory challenge and the protective capacity of ceftriaxone.

### Poly I:C–induced dendritic spine loss and ceftriaxone-mediated rescue

**Figure 3:**
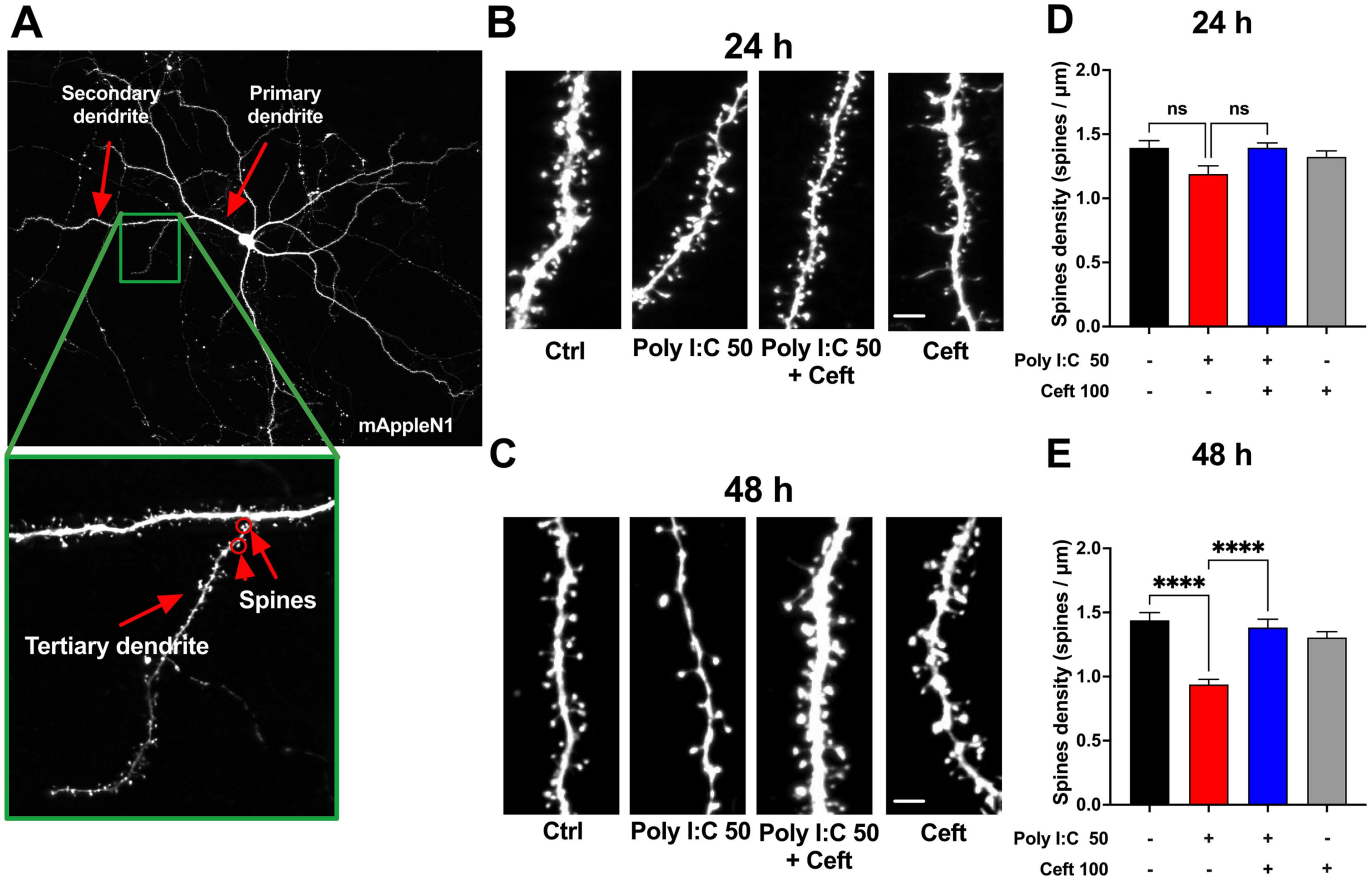
Effects of Poly I:C and ceftriaxone on dendritic spine density at 24 and 48 h Poly I:C treatment resulted in a time-dependent decrease in dendritic spine density, which was effectively prevented by ceftriaxone co-treatment. **(A)** Representative images of mApple N1-transfected neurons. Tertiary dendrites exceeding 60 μm in length were selected to ensure the maturity of the neurons analyzed. Spine density was assessed as the number of spines per micrometer across different treatment groups at 24 and 48 h post-administration. **(B,C)** Enlarged representative images of tertiary dendrites from mApple N1-transfected neurons under different treatment conditions at 24 h **(B)** and 48 h **(C)**. **(D,E)** Quantitative comparison of spine density on tertiary dendrites across treatment groups at 24 h **(D)** and 48 h **(E)**, expressed as spines per micrometer. Data are presented as mean ± SEM (24 h: *N* = 152; 48 h: *N* = 124). Statistical significance is indicated as follows: **** *p* < 0.0001, ns = not significant.

To assess Poly I:C’s impact on neuronal architecture, we quantified dendritic spine density in eGFP-labeled hippocampal neurons at 24 and 48 h post-treatment (Figure 3A). Dendritic spines underpin synaptic connectivity and plasticity, making their density a sensitive readout of neuronal health and function (Gipson and Olive, 2017). At 24 h, 50 μg/mL Poly I:C reduced spine density relative to controls, though not significantly (Figures 3B,D, ctrl). By 48 h, this spine loss reached statistical significance (*p* = 0.003, Figures 3C,E), indicating a time-dependent exacerbation of synaptic disruption. Remarkably, ceftriaxone co-treatment fully prevented Poly I:C–induced spine loss, restoring densities to control levels. Ceftriaxone alone had no effect on spine density at either time point.

### Synaptic plasticity under neuroinflammatory challenge: LTP impairment and ceftriaxone rescue

Long-term potentiation (LTP) is widely regarded as a cellular substrate of learning and memory, relying on sustained increases in synaptic strength (Korte and Schmitz, 2016). Because our earlier results showed that Poly I:C–induced inflammation disrupts glutamate clearance, glial morphology, and spine density, we next asked whether these structural changes translate into functional deficits in synaptic plasticity—and whether ceftriaxone can prevent them.

We transfected hippocampal neurons to express the calcium indicator GCaMP5g to monitor spine-specific calcium transients at 24 and 48 h post–Poly I:C treatment. After establishing baseline activity (−20 to −10 min pre-stimulation), we chemically induced LTP (cLTP) using glycine and strychnine, and then recorded calcium responses at 0, 10, and 40 min post-stimulation.

**Figure 4:**
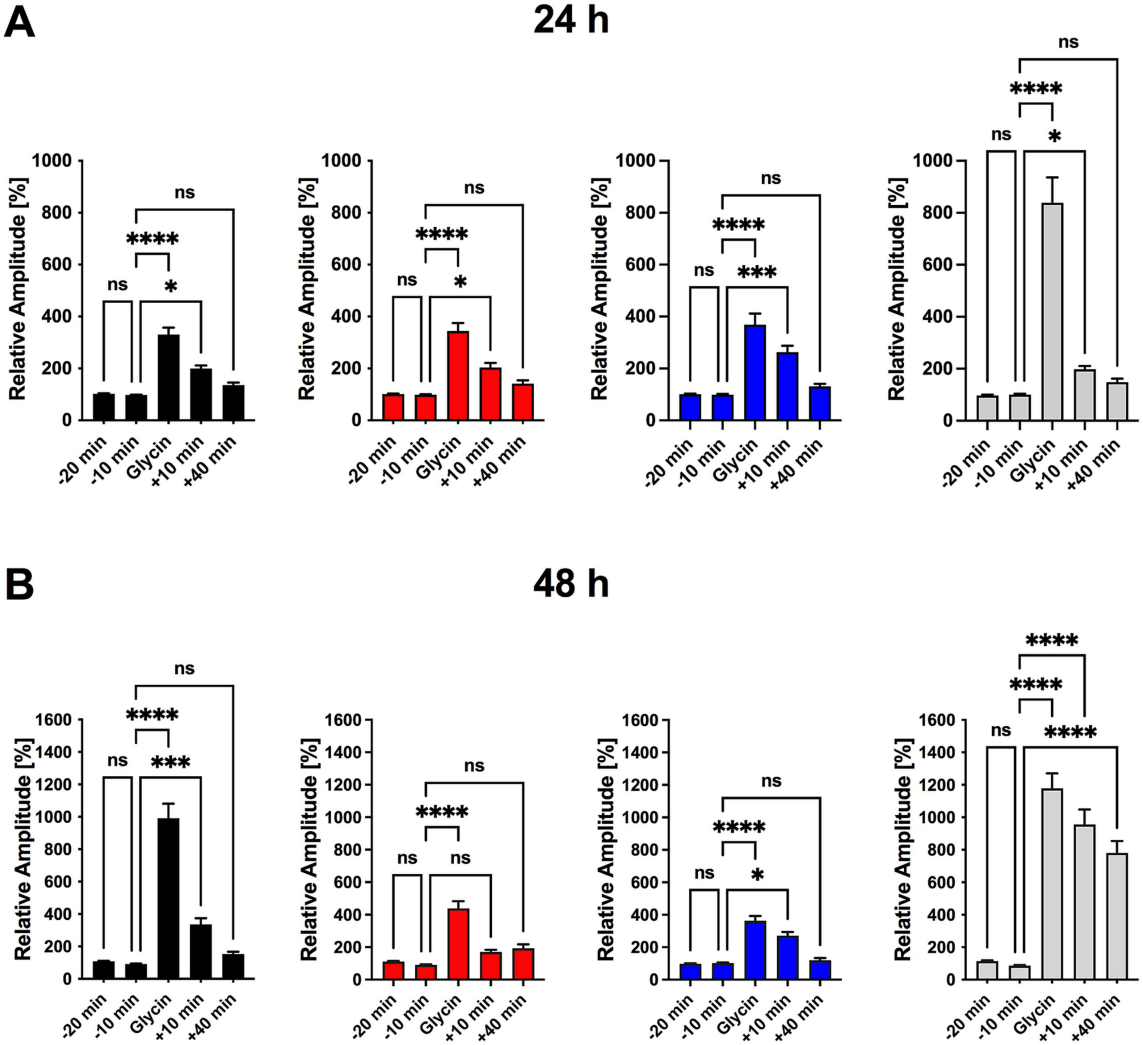
Ceftriaxone restores Poly I:C-induced impairment in LTP and enhances synaptic plasticity in hippocampal neuronal cultures. **(A)** Experimental timeline for a single imaging series. Transfected triple co-cultures were transferred to a recording chamber and perfused with room-temperature 1 × HBSS at 1 mL/min. Following a 20 min adaptation period, baseline calcium activity was recorded at *t* = −20 min and −10 min. Chemically induced long-term potentiation (cLTP) was then elicited by perfusing 1 × HBSS containing glycine and strychnine for 10 min. After cLTP induction, perfusion with 1 × HBSS resumed and calcium signals were recorded at *t* = 0, 10, and 40 min. **(B)** Normalized calcium-signal amplitudes measured 24 h after treatment. No significant differences were observed between *t* = −20 min and −10 min, confirming stable baseline activity (Figure 5B). All groups showed robust potentiation immediately after cLTP induction (*t* = 0), which persisted at *t* = 10 min; by *t* = 40 min post-washout, potentiation had returned to baseline in every group. **(C)** Normalized calcium-signal amplitudes measured 48 h after treatment. Ten minutes of glycine incubation induced significant cLTP in all groups. In the Poly I:C (50 μg/mL) group, signal amplitudes returned to baseline by *t* = 10 min, indicating impaired LTP maintenance. Co-treatment with ceftriaxone preserved potentiation at *t* = 10 min, rescuing the Poly I:C–induced deficit. Ceftriaxone alone produced the greatest enhancement of cLTP, with effects becoming more pronounced over time. Data are presented as mean ± SEM. *p < 0.05, ***p < 0.001, ****p < 0.0001; ns, not significant.

At 24 h, baseline calcium activity was stable across all groups, and cLTP induction produced comparable potentiation that waned by 40 min (Figure 4B). However, in Poly I:C-treated cultures at 48 h, the key 10-min potentiation phase was absent, indicating impaired LTP maintenance. Ceftriaxone co-treatment fully restored 10-min potentiation, while ceftriaxone alone further enhanced LTP magnitude and duration beyond control levels (Figure 4C). Spontaneous calcium transient frequency remained unchanged by any treatment (Supplementary Figure 2).

These findings demonstrate that Poly I:C–triggered neuroinflammation selectively compromises synaptic plasticity by specifically affecting LTP maintenance but not LTP induction, and that ceftriaxone not only rescues this deficit but may also bolster synaptic plasticity over time.

### Disruption and rescue of synaptic protein interactions in neuroinflammation

Understanding how neuroinflammatory insults impair synaptic plasticity requires examining key postsynaptic proteins. Ca^2+^/calmodulin-dependent protein kinase IIα (CaMKIIα) and the scaffold protein PSD-95 are central to LTP: CaMKIIα is activated by calcium influx, phosphorylates AMPA receptor subunits (e.g., GluA1), and promotes their synaptic insertion, while PSD-95 organizes these receptors and signaling complexes at the postsynaptic density (Shen and Meyer, 1999; Lisman et al., 2002; Sun and Turrigiano, 2011). Co-localization of CaMKIIα with PSD-95 is therefore a hallmark of a functional synaptic machinery; disruption of this association can weaken LTP and compromise cognitive processes.

**Figure 5:**
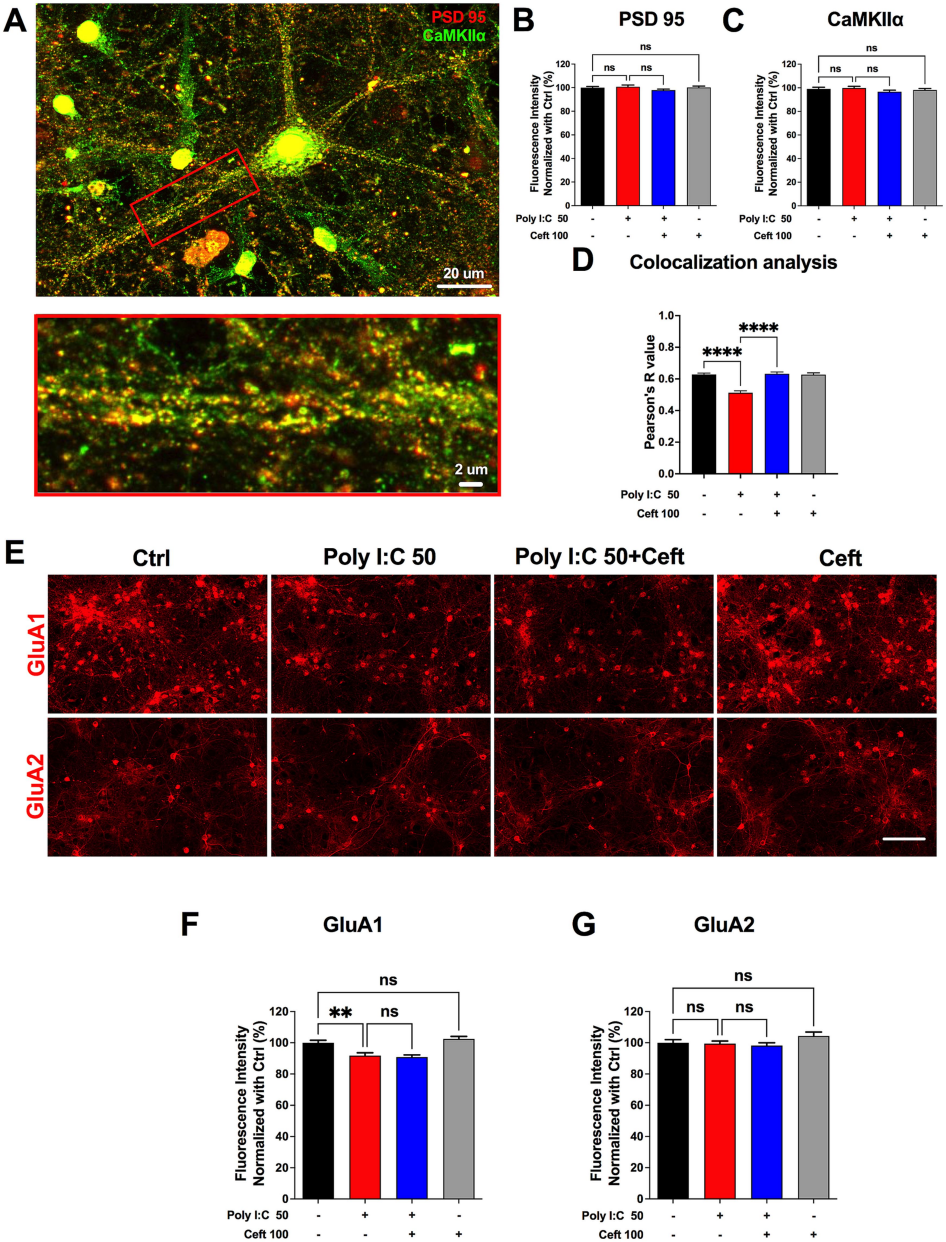
Ceftriaxone rescues the reduced colocalization of PSD-95 and CaMKIIα induced by Poly I:C. **(A)** Representative image used for colocalization analysis. The red box denotes the zoomed-in ROI highlighting a primary dendrite of a single neuron. Scale bars: 20 μm (overview) and 2 μm (zoom). **(B,C)** Quantification of normalized expression levels at 48 h post-treatment: PSD-95 (**B**, red) and CaMKIIα (**C**, green). **(D)** Pearson’s R coefficients for colocalization of CaMKIIα and PSD-95 within primary dendrites. **(E)** Representative immunocytochemical staining of AMPA-receptor subunits GluA1 and GluA2. Scale bar: 100 μm. **(F,G)** Normalized expression levels of GluA1 **(F)** and GluA2 **(G)** at 48 h post-treatment. Data are presented as mean ± SEM. **p < 0.01, ****p < 0.0001; ns, not significant.

We first measured total CaMKIIα and PSD-95 expression and found no changes after Poly I:C or ceftriaxone treatment (Figures 5A–C), indicating that altered protein abundance does not underlie LTP deficits. We then assessed their synaptic colocalization by dual immunofluorescence (CaMKIIα in green, PSD-95 in red) and computed Pearson’s correlation within dendritic regions of interest (Figure 5A). Poly I:C treatment significantly reduced CaMKIIα/PSD-95 colocalization (Figure 5D), reflecting impaired postsynaptic complex assembly. Strikingly, ceftriaxone co-treatment fully restored this colocalization, suggesting a mechanism for its preservation of synaptic plasticity.

Changes in the composition and surface expression of AMPA receptor subunits critically shape synaptic transmission and plasticity. GluA1-containing receptors are dynamically trafficked into the postsynaptic membrane during LTP induction, enhancing synaptic strength, whereas GluA2 confers calcium impermeability and stabilizes receptor assemblies (Heine et al., 2008). Thus, selectively assessing GluA1 and GluA2 levels reveals how neuroinflammatory insults or therapeutic interventions alter receptor composition, calcium signaling, and the capacity for synaptic potentiation. Poly I:C markedly decreased GluA1 levels, an effect not rescued by ceftriaxone, whereas GluA2 expression remained unchanged across all conditions (Figures 5E–G).

These data indicate that Poly I:C disrupts the spatial organization of critical synaptic proteins without altering their total expression, and that ceftriaxone can re-establish proper CaMKIIα/PSD-95 interactions—potentially underpinning its restoration of LTP in an inflammatory context.

### Poly I:C–induced metabolic dysregulation and its rescue by ceftriaxone

Having demonstrated that Poly I:C provokes neuroinflammation, downregulates GLT-1, and impairs synaptic function, we next interrogated its effects on cellular metabolism. The glutamate–glutamine cycle bridges neurotransmitter balance with the tricarboxylic acid (TCA) cycle and is vital for neuronal and astrocytic health. Disruption of this axis can precipitate excitotoxicity and energy failure. By profiling intracellular amino acids and TCA intermediates, we aimed to define the metabolic consequences of Poly I:C exposure and to test whether ceftriaxone restores homeostasis—thereby illuminating its neuroprotective mechanisms and identifying potential metabolic biomarkers.

**Figure 6:**
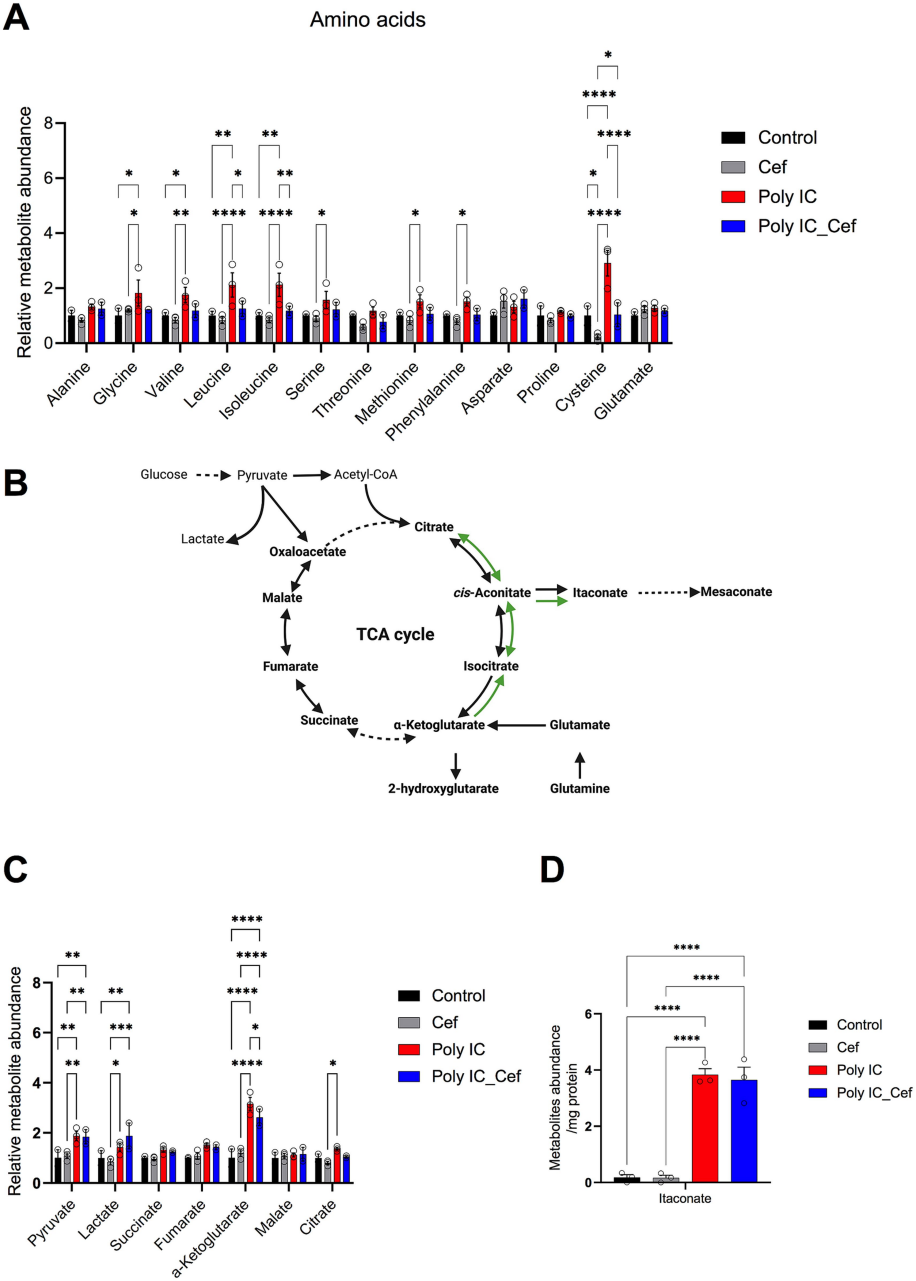
Poly I:C and ceftriaxone modulate intercellular metabolite levels in the tri-culture system. **(A)** Levels of intercellular amino acids in the tri-culture system after 48 h of Poly I:C stimulation (50 μg/mL) and ceftriaxone treatment (100 μM). Poly I:C significantly increased intercellular amino acid levels, while ceftriaxone restored them to baseline. **(B)** Poly I:C increased tricarboxylic acid (TCA) cycle-related metabolites, particularly α-ketoglutarate, while ceftriaxone reversed these changes. **(C)** Poly I:C increased itaconate levels. Data represent the relative abundance of metabolites identified through GC–MS, presented as mean ± SEM from 3 biological replicates. Statistical significance was determined using One-way or Two-way ANOVA (*p < 0.05, **p < 0.01, ***p < 0.001, ****p < 0.0001).

After 48 h of treatment with 50 μg/mL Poly I:C, with or without 100 μM ceftriaxone, we quantified intracellular metabolites in our neuron–astrocyte–microglia co-cultures (Figure 6A). Poly I:C significantly elevated several amino acids whereas ceftriaxone co-administration normalized these levels. Intracellular levels of glutamate and aspartate—the primary substrates of the GLT-1 (EAAT2/SLC1A2) transporter—remained unchanged in Poly I:C treated conditions. This suggests that the observed amino acid alterations might be independent of GLT-1-mediated transport activity. Ceftriaxone alone had no appreciable effect on baseline amino acid pools.

We then quantified TCA cycle–related metabolites (Figures 6B–D). Poly I:C markedly increased itaconate—an immunomodulatory TCA derivative (Michelucci et al., 2013)—and other intermediates, especially α-ketoglutarate (Figure 6D). Co-treatment with ceftriaxone decreased α-ketoglutarate levels and partially reversed elevations in other TCA metabolites.

Together, these data show that Poly I:C–driven neuroinflammation modulates amino acid homeostasis and rewires TCA cycle metabolism, while ceftriaxone partially rebalances these metabolic pathways, supporting neuronal and glial bioenergetics in an inflammatory milieu. By integrating these metabolic insights with our structural and functional findings, we achieve a holistic understanding of Poly I:C pathology and ceftriaxone’s multifaceted neuroprotection—paving the way for targeted therapies in inflammatory neurodegenerative disorders.

### Mendelian randomization (MR)-causal associations of genetically predicted plasma amino acid levels with 5 neurodegenerative diseases

The central nervous system (CNS) is essential for maintaining metabolic homeostasis, including the synthesis, utilization, and recycling of amino acids. The glutamate-glutamine cycle is crucial for balancing neurotransmitters and supporting energy metabolism in neurons and astrocytes. Poly I:C-induced neuroinflammation disrupts CNS metabolism, leading to systemic metabolic disturbances. For example, decreased GLT-1 expression impairs glutamate uptake, alters amino acid flux (Rothstein et al., 1996) might therefore affect plasma amino acid levels (Quintero Escobar et al., 2021). Because plasma amino acid levels integrate both central and peripheral metabolism, they hold promise as biomarkers for neurodegenerative diseases.

To test whether these metabolic shifts contribute causally to neurodegeneration, we complemented our *in vitro* data with a GWAS-based Mendelian randomization analysis (Zheng et al., 2020). Using genetic variants as instruments, we evaluated associations between nine plasma amino acids and the risk of five major neurodegenerative disorders. We identified significant causal links, demonstrating that inflammation-driven amino acid imbalance in our cellular model echoes systemic metabolic alterations in patients. These results not only validate the clinical relevance of our findings but also strengthen the rationale for targeting amino acid homeostasis—such as through ceftriaxone-mediated restoration of GLT-1—to forestall neuronal degeneration.

**Figure 7:**
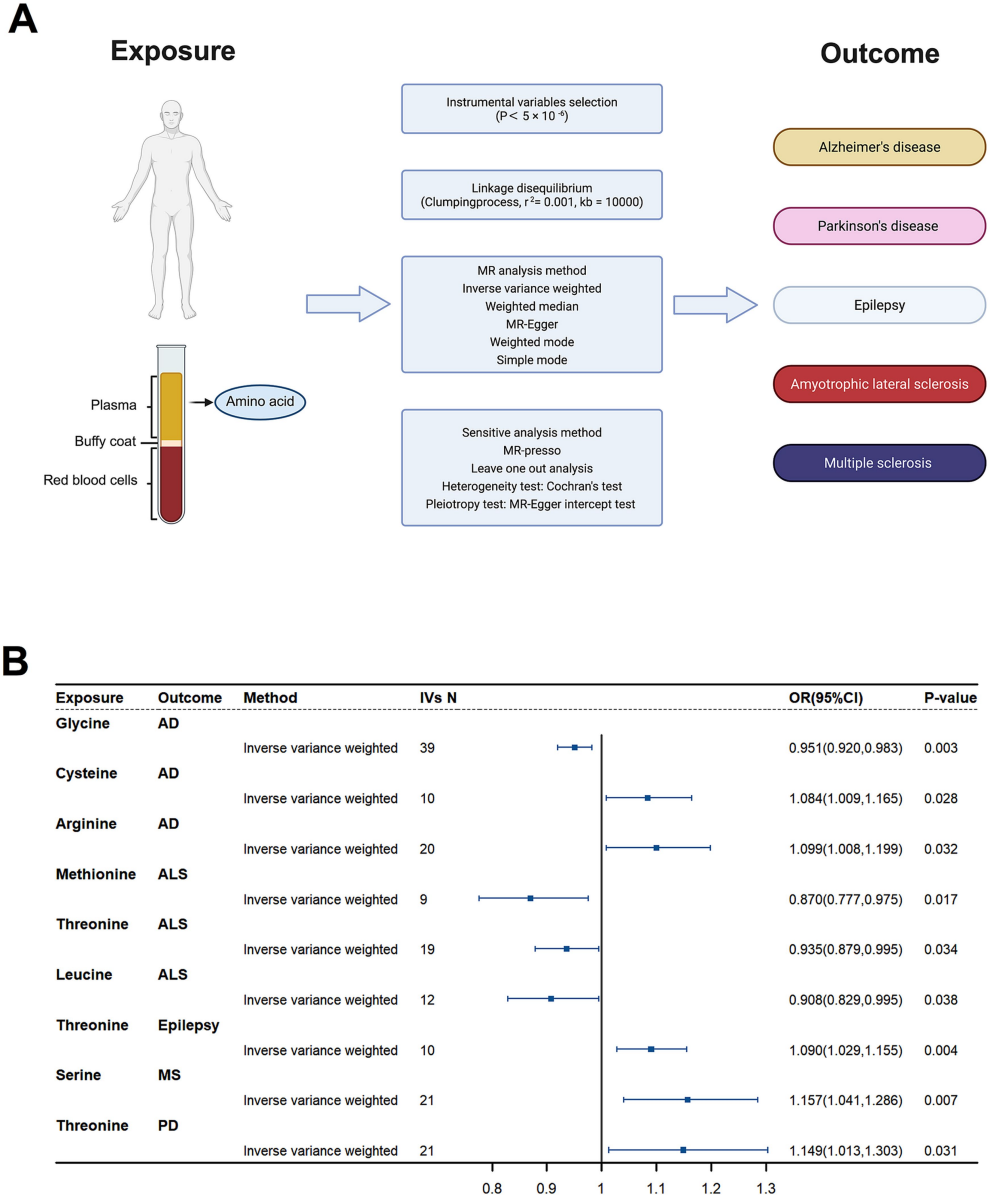
Causal effects of plasma amino acids on neurodegenerative disease risk: a Mendelian Randomization study. **(A)** Schematic of the Mendelian Randomization (MR) study design. Genetic instruments for nine plasma amino acids were used to assess their causal impact on five neurodegenerative diseases. **(B)** Forest plot of inverse-variance weighted (IVW) MR estimates for the nine amino acids. Shown are odds ratios (ORs) and 95% confidence intervals for associations that reached nominal significance (*p* < 0.05).

To define causal relationships between plasma amino acid levels and neurodegenerative disease risk, we performed a two-sample Mendelian randomization (MR) analysis (Figure 7A). Using the inverse-variance weighted (IVW) method, we found that genetically higher concentrations of five amino acids were associated with increased risk of specific neurodegenerative disorders, while genetically lower levels of four amino acids corresponded to reduced risk (Figure 7B). These findings implicate distinct amino acids as causal drivers or protectors in neurodegeneration and reinforce the utility of amino acid metabolism both as a biomarker and as a therapeutic target in these diseases.

## Discussion

In this study, we demonstrate that ceftriaxone confers broad neuroprotection against Poly I:C–induced neuroinflammation in an in vitro co-culture model. Its multifaceted actions include upregulation of astrocytic GLT-1, preservation of synaptic integrity and plasticity, and normalization of inflammation-induced metabolic reprogramming.

### Glutamate transporter regulation

Downregulation of GLT-1 is a well-recognized mediator of glutamate excitotoxicity in neurodegenerative diseases (Rothstein et al., 2005; Tai et al., 2019). In our model, Poly I:C triggered a dose-dependent decline in GLT-1 expression, which ceftriaxone fully restored at both 24 and 48 h. By rescuing astrocytic glutamate clearance, ceftriaxone likely prevents the accumulation of extracellular glutamate that drives neuronal injury under inflammatory conditions, as previously shown (Rothstein et al., 2005).

Interestingly, we observed a dose-dependent divergence in astrocytic glutamate transporter regulation: while high-dose Poly I:C downregulated GLT-1, low-dose exposure selectively upregulated GLAST (EAAT1/SLC1A3). This increase in GLAST was not reversed by ceftriaxone, which instead mildly reduced GLAST expression when applied alone. These findings suggest that GLAST may serve as a compensatory glutamate buffer under low-grade inflammatory stress, distinct from the GLT-1–mediated response to severe neuroinflammation. Such a role for GLAST in early reactive astrocyte responses has been suggested in studies showing regulation of GLAST by astrocyte-derived growth factors and oxidative stress (Swanson et al., 1997). The lack of ceftriaxone effect on GLAST aligns with its known specificity for GLT-1 transcriptional upregulation via NF-κB and CREB-dependent pathways (Rothstein et al., 2005). Moreover, the mild reduction of GLAST by ceftriaxone alone could indicate a shift toward GLT-1–dominated glutamate handling, possibly reflecting a more mature or energy-efficient astrocytic phenotype, as suggested by the dynamic regulation of GLT-1 surface mobility and synaptic coordination (Murphy-Royal et al., 2015). Whether such shifts have functional consequences for glutamate homeostasis or synaptic signaling in different inflammatory contexts remains to be explored.

### Modulation of glial activation and cytokine responses

Beyond its glutamate-modulating action, ceftriaxone reduced microglial hypertrophy and helped maintain connexin-43–mediated astrocytic coupling—both key factors in dampening neuroinflammation (Lim et al., 2021). We also observed that Poly I:C induced mitochondrial swelling in GFAP^+^ astrocytes (COX4 particle enlargement), whereas ceftriaxone reversed these mitochondrial alterations, suggesting improved oxidative phosphorylation and resilience under inflammatory stress. Reactive glial hypertrophy is functionally coupled to excitotoxic stress: when astrocytic GLT-1 is reduced, enlarged/reactive astrocytes clear glutamate less efficiently, promoting synaptic spillover and activation of extrasynaptic NMDARs that compromise neuronal survival (Danbolt, 2001; Hardingham and Bading, 2010). Concomitant changes in connexin-43 coupling and mitochondrial stress further weaken K^+^/neurotransmitter buffering and network homeostasis (Rouach et al., 2008; Verkhratsky and Nedergaard, 2018). Microglial hypertrophy amplifies this milieu via cytokine/ATP signaling that depresses astrocytic uptake and destabilizes synapses (Tilleux and Hermans, 2007; Kettenmann et al., 2011). By restoring GLT-1 and preserving astrocytic coupling, ceftriaxone likely interrupts this glia–glutamate–excitotoxicity loop and thereby protects dendritic structure and cLTP maintenance (Rothstein et al., 2005; Murphy-Royal et al., 2015).

An unexpected finding in our model was that, despite clear reductions in microglial hypertrophy and preservation of astrocytic coupling, ceftriaxone did not prevent the Poly I:C–induced rise in TNF-α, IL-6, or IL-10. This contrasts with *in vivo* reports—particularly in traumatic brain injury models—where ceftriaxone treatment was associated with lower microglial cytokine expression (Lim et al., 2021). Several factors may contribute to this discrepancy: (1) the temporal and contextual specificity of ceftriaxone’s effects, (2) differences in the injury model (viral mimic vs. mechanical trauma), and (Wagner et al., 2011) limitations of bulk cytokine analysis in mixed cultures. It is plausible that ceftriaxone modulates inflammatory signaling at the level of cell morphology and metabolic state rather than transcriptionally suppressing cytokines across the board. These considerations suggest that ceftriaxone’s primary neuroprotective mechanism may lie in limiting the morphological and functional hallmarks of glial activation and restoring metabolic and synaptic homeostasis, rather than broadly inhibiting cytokine release. Future studies should compare time-dependent cytokine profiles in vivo and *in vitro* and employ single-cell approaches to disentangle how ceftriaxone modulates glial signaling pathways under different injury paradigms. Cytokine profiling was limited to TNF-α, IL-6, and IL-10; broader panels (e.g., IL-1β, IFN-γ, MCP-1) will be examined in future work to further clarify the effect of ceftriaxone.

### Synaptic protection and plasticity

Our analysis of synaptic structure and function showed that Poly I:C reduced dendritic spine density and selectively impaired the maintenance phase of chemically induced long-term potentiation (cLTP), without affecting LTP induction. Ceftriaxone co-treatment prevented spine loss and fully restored 10-min cLTP retention. Mechanistically, Poly I:C disrupted the colocalization of CaMKIIα with PSD-95—a marker of functional postsynaptic complexes—while ceftriaxone reestablished this interaction. Although Poly I:C also downregulated GluA1 AMPA receptor subunits, ceftriaxone did not rescue GluA1 levels, indicating that its LTP-preserving effects may rely primarily on restoring synaptic protein organization rather than receptor abundance. The restored CaMKIIα–PSD-95 interaction implies that ceftriaxone helps preserve postsynaptic signaling scaffolds essential for cLTP maintenance, highlighting its role in sustaining synaptic signaling architecture even under persistent inflammatory stress. These findings align with reports that ceftriaxone inhibits microglial phagocytosis of synaptic elements and bolsters GLT-1–mediated glutamate clearance to preserve synaptic integrity (Liu et al., 2023; Jiang et al., 2024).

### Immunometabolic reprogramming and mitochondrial restoration

Poly I:C exposure for 48 h induced metabolic reprogramming in neuron-astrocyte-microglia co-cultures indicative of an inflammatory response. This was evidenced by a significant accumulation of itaconate, an immunometabolite produced by IRG1-mediated decarboxylation of cis-aconitate in activated myeloid cells (Michelucci et al., 2013).

Itaconate accumulation inhibits succinate dehydrogenase, perturbing mitochondrial respiration, and modulating redox balance (Ye et al., 2024). Beyond its role in inflammation, itaconate has antiviral effects in a model of influenza A virus (IAV) infection (Sohail et al., 2022). Its accumulation also contributes to the host’s ability to counteract viral infections, highlighting its potential as a therapeutic target in inflammatory and infectious diseases. Notably, intracellular glutamate and aspartate—the primary substrates of the GLT-1 transporter—remained unchanged across the Poly I:C condition. This observation suggests that the alterations in amino acid levels are independent of GLT-1-mediated transport activity. However, a broad set of other amino acids—including glycine, valine, leucine, isoleucine, serine, methionine, phenylalanine, and cysteine—were significantly elevated. Simultaneously, the tricarboxylic acid (TCA) cycle intermediate α-ketoglutarate accumulated, suggesting impact on mitochondrial metabolism. Ceftriaxone co-treatment effectively reversed these metabolic derangements of amino acid levels. By upregulating astrocytic GLT-1, ceftriaxone enhances glutamate uptake and its controlled conversion via glutamate dehydrogenase into α-ketoglutarate, thereby restoring smooth carbon flux through the TCA cycle. This normalization of metabolic pathways contributes to the rescue of mitochondrial morphology and function—evidenced by the reversal of Poly I:C–induced mitochondrial swelling in GFAP^+^ astrocytes. Finally, the normalization of several amino acids suggests that restored glial-neuronal metabolic coupling prevents both excessive proteolytic release and impaired amino acid homeostasis.

## Conclusion and implications

Together, these data position ceftriaxone not only as an excitotoxicity blocker via GLT-1 upregulation, but also as a potent corrector of inflammation-driven metabolic imbalance. By influencing TCA cycle homeostasis, and normalizing amino acid pools, ceftriaxone may interrupt the immunometabolic feed-forward loop that links neuroinflammation to synaptic dysfunction and long-term neurodegeneration.

These *in vitro* findings resonate with our Mendelian randomization results linking genetically determined amino acid levels to neurodegenerative disease risk: elevated cysteine and arginine increase susceptibility, whereas higher glycine, threonine, methionine, and leucine are protective. By restoring both TCA cycle homeostasis and balanced amino acid metabolism, ceftriaxone may thus interrupt the vicious cycle of inflammatory metabolic dysregulation that contributes to synaptic dysfunction and chronic neurodegeneration.

Taken together, our findings identify ceftriaxone as a multifaceted agent that interrupts key pathological loops in neuroinflammation by preserving glutamate clearance, limiting glial activation, sustaining synaptic plasticity, and reestablishing metabolic homeostasis. These properties support its potential as an early intervention strategy for infection-related and chronic neurodegenerative diseases (Jurgens et al., 2012; Heneka et al., 2015; Liddelow et al., 2017; Hosseini et al., 2018).

Our data nominate GLT-1 restoration as a tractable axis to blunt virus-triggered neuroinflammation and secondary synaptic/metabolic injury. In a clinical trajectory, the most plausible window is early post-infection or during subacute neuroinflammatory states, where preserving astrocytic uptake and synaptic scaffolds could limit downstream cognitive decline. While ceftriaxone offers a well-characterized, CNS-penetrant tool compound, real-world deployment must balance antibiotic stewardship and dosing constraints; thus, non-antibacterial GLT-1 enhancers or derivatives may provide safer chronic strategies. Our Mendelian-randomization link between amino-acid profiles and neurodegeneration risk further suggests metabolic biomarkers (e.g., branched-chain amino acids, itaconate/TCA signatures) for patient stratification and pharmacodynamic readouts. Prospective studies should pair GLT-1 target engagement (e.g., PET or CSF markers), cognitive endpoints, and safety monitoring to test whether early GLT-1 augmentation interrupts the glia–excitotoxic–metabolic feed-forward loop.

Limitations of our study include the use of an in vitro model that does not fully recapitulate *in vivo* complexity and the inability to distinguish metabolite changes among specific cell types. Future work should validate these mechanisms in animal models and patient tissues, explore long-term efficacy and safety of ceftriaxone, and investigate potential synergistic therapies that target complementary pathways.

## Materials and methods

### Animals

Primary hippocampal neurons and pure microglial cells were isolated from C57BL/6 mice. All animal procedures were conducted in compliance with ethical guidelines and were approved by the Animal Welfare Representative of TU Braunschweig and the Landesamt für Verbraucherschutz und Lebensmittelsicherheit (LAVES) in Oldenburg, Germany (Genehmigungsnummer §4 (09.22) TSB TU BS). Mice were housed under a 12-h light/dark cycle at a temperature of 22 °C. They were maintained in standardized cages with unrestricted access to food and water (ad libitum) to ensure optimal living conditions.

### Preparation of primary embryonic hippocampal culture

Primary hippocampal neurons were isolated from embryonic day 17.5 (E17.5) C57BL/6 mice of mixed genders. Following immediate extraction, the embryos were dissected to isolate the hippocampi, which were then transferred to ice-cold 1× GBSS solution supplemented with glucose. The hippocampal tissue was enzymatically digested using 1 mL of Trypsin/EDTA solution at 37 °C for 25 min. Digestion was halted by washing the tissue five times with serum-containing medium. Subsequently, the hippocampi were mechanically dissociated using a fire-polished Pasteur pipette. The triturated cell suspension was centrifuged at 1,500 rpm for 5 min, resuspended in 1 mL of NB+ culture medium, and counted using a Neubauer chamber. Cells were plated at a density of 70,000 cells per well on poly-L-lysine-coated glass coverslips in 24-well plates. Cultures were maintained at 37 °C in a humidified incubator with 5% CO_2_. The culture media (100 μL per well) were refreshed weekly until Day 21 when the neurons and astrocytes were ready for experimental use.

### Preparation of primary microglia culture

Primary microglia were harvested from postnatal day 3–5 (P3–P5) C57BL/6 mouse pups. Following decapitation, brains were promptly collected and placed in cold 1× HBSS solution. The meninges and hippocampi were removed, and only cortical tissue was retained in a new petri dish containing 10 mL of 1× HBSS. The cortex was transferred to sterile 15 mL conical tubes and centrifuged at 400×*g* for 5 min at 4 °C. The cell pellet was passed through a 100 μm cell strainer, centrifuged again at 400×*g* for 5 min at 4 °C, and resuspended in fresh cold 1× HBSS at a ratio of one cortex per milliliter. Equal volumes (1 mL) of the cell suspension were seeded into each T-75 flask and incubated at 37 °C in a humidified incubator with 10% CO_2_ and 95% humidity. After 2–3 days, the culture medium was completely replaced with fresh DMEM supplemented with 10% fetal calf serum (FCS) and antibiotics (P/S). The medium was refreshed with 10 mL per flask weekly until the microglia were harvested between Days 14 and 17.

### Establishment of the triple co-culture

To create triple co-cultures, pure microglia were then isolated at 14 days *in vitro* (Cordes et al., 2020) and seeded onto the neuron-astrocyte cultures at DIV 21. Microglial flasks were placed on a shaker at 220 rpm for 2 h at 37 °C to detach the cells. The supernatant, containing microglia, was collected into 50 mL conical tubes without disturbing the astrocyte layer. The collected media were centrifuged at 400×*g* for 5 min at room temperature, and the supernatant was aspirated. The resulting microglial pellet was resuspended in NB + medium, counted using a Neubauer chamber, and plated onto the DIV 21 embryonic hippocampal culture at a density of 30,000 cells per well in 24-well plates. The co-cultures were maintained for an additional 48 h at 37 °C, 5% CO_2_, and 99% humidity before being used for experimental procedures.

### Transfection of primary embryonic hippocampal cultures

To analyze spine density, primary hippocampal neurons were transfected with recombinant DNA (mApple N1) using Lipofectamine® 2000 (Thermo Fisher Scientific) according to the manufacturer’s protocol. To visualize spontaneous calcium transients, primary neurons were transfected with the expression plasmid for GCaMP5g (Addgene, Cat# 31788). Briefly, Neurobasal (NB-) medium without supplements was pre-warmed to 37 °C in an incubator maintained at 5% CO_2_ and 99% humidity. For each well of a 24-well plate, two separate mixtures were prepared: 1. DNA Mixture: 50 μL NB− medium + 1 μL mApple N1 plasmid (1 μg/μl); 2. Transfection Reagent Mixture: 50 μL NB− medium + 2 μL Lipofectamine® 2000.

Each mixture was incubated separately at room temperature for 5 min. Subsequently, the DNA and transfection reagent mixtures were combined and incubated at room temperature for an additional 20 min to allow complex formation. The existing cell culture medium in each well was replaced with 300 μL of pre-warmed NB− medium to maintain cell viability during transfection. Next, 100 μL of the plasmid-Lipofectamine® 2000 complex was added dropwise to each well. The 24-well plates were gently rocked back and forth to ensure thorough mixing of the medium and transfection complexes. Transfection proceeded with a 45-min incubation at 37 °C, 5% CO_2_, and 99% humidity. Following incubation, the transfection medium was replaced with the original conditioned NB+ medium to support cell recovery and growth. To obtain high-quality neuronal images for spine counting, transfection with mApple N1 plasmid was performed 24–48 h prior to cell fixation.

### Polyinosinic:polycytidylic acid (Poly I:C) and ceftriaxone administration

Polyinosinic:polycytidylic acid (Poly I:C; Sigma-Aldrich, P1530) was utilized to induce neuroinflammation in the triple co-cultures, while ceftriaxone (Supelco, PHR 1382) served as a therapeutic agent to counteract Poly I:C-induced GLT-1 downregulation. Poly I:C lyophilized powder was dissolved in sterile Milli-Q water to prepare a 10 mg/mL stock solution. To prevent freeze–thaw cycles, the stock was aliquoted into 1.5 mL Eppendorf tubes at 50 μL per tube and stored at −20 °C. Similarly, ceftriaxone sodium powder was dissolved in sterile Milli-Q water to prepare a 0.5 M stock solution, aliquoted at 50 μL per tube, and stored at −20 °C. On the day of treatment, stock solutions were diluted into fresh NB+ medium to prepare the desired treatment concentrations.

Cell cultures were divided into six groups: Control (Ctrl), Poly I:C 25 μg/mL, Poly I:C 50 μg/mL, Ceftriaxone 100 μM, Poly I:C 25 μg/mL + Ceftriaxone 100 μM, and Poly I:C 50 μg/mL + Ceftriaxone 100 μM. Treatments were administered at 24 h and 48 h post-Poly I:C exposure to assess both early and sustained effects of the interventions.

### Cytokine assessment

The expression levels of the cytokines TNF-α, IL-6, and IL-10 were quantified using ELISA kits (R&D Systems: TNF-α DY410-05, IL-6 DY406-05, IL-10 DY417-05). No other cytokines were measured. Culture medium was collected from each well 24 h post-treatment, immediately frozen in liquid nitrogen, and stored at −70 °C until analysis. All procedures were performed according to the manufacturer’s protocol.

Briefly, detection plates were coated overnight with 100 μL of the respective diluted capture antibody. The following day, plates were washed three times with washing buffer. Next, 100 μL of samples or standards, prepared as per the manufacturer’s instructions, were added to each well and incubated for 2 h. After incubation, 100 μL of the detection antibody was added and incubated for an additional 2 h. Following three more washes, 100 μL of substrate solution was added to each well and incubated in the dark for 20 min. The reaction was terminated by adding 50 μL of stop solution to each well. Absorbance at 450 nm was measured using an Epoch microplate reader (BioTek). Data were analyzed using Gen5 software (BioTek).

### Immunocytochemistry

Immunocytochemistry was performed to identify and visualize different cell types and to quantify various proteins through immunofluorescence. After fixation with PFA, combination cultures were first blocked in a solution containing 0.2% Triton X-100, 1.5% goat serum, and/or 1.5% donkey serum in 1× PBS for 1 h at room temperature (RT). Following blocking, the cultures were incubated overnight at 4 °C with primary antibodies diluted in the blocking solution. The primary antibodies used were CaMKIIα (goat, 1:750, Abcam, ab111890), COX4 (rabbit, 1:500, Synaptic Systems, AB_2620041), Connexin 43 (rabbit, 1:500, Sigma-Aldrich, C6219), EAAT1/GLAST-1/SLC1A3 (rabbit, 1:500, Novus Biologicals, NB100-1869), EAAT2/GLT-1 (rabbit, 1:500, Novus Biologicals, NBP1-20136), GFAP (mouse, 1:1000, Sigma-Aldrich, G3893), GluR1 (GluA1) (guinea pig, 1:400, Alomone Labs, AGP-009), GluR2 (GluA2) (rabbit, 1:400, Alomone Labs, AGC-005), IBA-1 (mouse, 1:1000, Synaptic Systems, 234011), PSD-95 (mouse, 1:750, Novus Biologicals, NB300-556), and MAP2 (chicken, 1:1000, Synaptic Systems, 188006).

The following day, cultures were washed six times with washing buffer, each wash lasting 5 min. Secondary antibodies conjugated with fluorophores were then applied at twice the dilution of the corresponding primary antibodies and incubated for 2 h at RT. The secondary antibodies included CyTM2-conjugated AffiniPure Goat Anti-Rabbit IgG (H + L) (Jackson ImmunoResearch, AB_2338021), Cy™3-conjugated AffiniPure Donkey Anti-Goat IgG (H + L) (Jackson ImmunoResearch, AB_2340411), CyTM3-conjugated AffiniPure Goat Anti-Rabbit IgG (H + L) (Jackson ImmunoResearch, AB_2338006), Cy™3-conjugated AffiniPure F(ab’)₂ Fragment Donkey Anti-Guinea Pig IgG (H + L) (Jackson ImmunoResearch, AB_2340461), Cy™5-conjugated AffiniPure Donkey Anti-Chicken IgY(H + L) (Jackson ImmunoResearch, AB_2340365), DyLight™ 405-conjugated AffiniPure Goat Anti-Mouse IgG (H + L) (Jackson ImmunoResearch, AB_2338786), and Alexa Fluor® 647-conjugated AffiniPure Goat Anti-Mouse IgG (H + L) (Jackson ImmunoResearch, AB_2338912).

After secondary antibody incubation, cultures were washed five times with washing buffer, each wash lasting 5 min. Nuclei were counterstained by incubating the cultures with 4′,6-diamidino-2-phenylindole (DAPI) at a dilution of 1:1,000 in 1× PBS for 5 min, followed by five additional washes. Coverslips were then mounted using Fluoro-gel medium (Electron Microscopy Sciences). All incubation steps were performed on a shaker at RT unless otherwise specified.

### Quantitative immunofluorescence analysis

To quantitatively assess the expression levels of GFAP, GLT-1, GLAST, CX43, GluA1, and GluA2, immunofluorescence staining was performed at both 24 and 48 h post-treatment using the respective primary antibodies as detailed in the immunocytochemistry section. Fluorescence images were acquired using a ZEISS Apotome 2 fluorescence microscope equipped with a Plan-Apochromat 20×/0.8 objective (ZEISS). Each standard image comprised z-stacks captured at 1 μm intervals to ensure comprehensive visualization of the microglia and astrocytes. Images were randomly selected to eliminate selection bias. Fluorescence intensity was quantified using ImageJ software (version 1.52) and normalized to control groups to facilitate accurate comparison between different treatment groups.

### Quantification of dendritic spine density

To quantify spine density, primary hippocampal neurons were transfected with an expression plasmid carrying mApple (as described in the “Transfection of Primary Hippocampal Neurons” section). Spine density measurements were conducted at both 24 and 48 h post-treatment.

Fluorescence images for spine density analysis were acquired using a ZEISS AxioImager2 upright fluorescence microscope equipped with an Apotome 2 and a Plan-Apochromat 63×/1.4 Oil objective (ZEISS, 420780-9900). Each standard image was captured as a series of z-stacks with 0.5 μm intervals, resulting in a pixel size of 0.093 μm at 63× magnification. Neurons were randomly selected for imaging, and only tertiary dendrites longer than 60 μm were analyzed.

Dendritic spines and their corresponding dendrites were manually delineated and selected using ImageJ software (version 1.52). Spine density was calculated as the number of spines per micrometer of dendrite length. This quantitative analysis allowed for the comparison of spine density between different treatment groups at the specified time points.

### cLTP induction and calcium imaging

Calcium imaging experiments were conducted on primary cultures at DIV 24–25 using either an Olympus BX61WI upright fluorescence microscope equipped with a 40× Plan-Apochromat (40×/0.7 NA) objective and a Hamamatsu ORCA-R2 CCD camera or an Olympus LSM FluoView FVMPE-RS System equipped with an Insight 2-Photon laser (Spectra-Physics) and a 25× objective (XLPLN25×WMP2, NA 1.05). Time-lapse recordings were acquired utilizing a pixel size of 0.093 μm and an acquisition time of 67 ms, with FITC (494 nm) as the excitation source. Each 100-s recording comprised 500 consecutive images, captured at 5 Hz intervals. Prior to imaging, transfected culture coverslips were transferred to a recording chamber containing room-temperature 1× HBSS and allowed to acclimate for 20 min under continuous perfusion with 1 mL/min of 1× HBSS provided by a peristaltic pump. Baseline Activity: Following the acclimation period, two spontaneous activity recordings were obtained at intervals of 10 min (t − 20 and t − 10). cLTP Induction: Chemical long-term potentiation (cLTP) was induced by applying 1× HBBS containing 1 mM glycine (AppliChem) and 1 μM strychnine hydrochloride (Sigma) for 10 min to block endogenous glycine receptors. Subsequently, continuous perfusion with 1× HBBS was maintained to wash out the stimulation solution, allowing for the acquisition of three additional recordings (t 0, t 10, and t 40 min).

For each imaged neuron, nine dendritic spines were selected as regions of interest (ROIs), and one background area was designated for reference. Fluorescence intensity was measured and background-corrected relative fluorescence (ΔF/F₀) was calculated using the formula ΔF/F0 = [(F − B) − (F0 − B0)]/(F0 − B0) via Matlab (R2020b, MathWorks). After noise filtering, fluorescence maxima of each calcium transient peak were identified. The amplitude and frequency of calcium transients were averaged across the imaging period for comparison. Signal amplitude at each time point was normalized to baseline activity.

### CaMKIIα and PSD95 colocalization

To evaluate the colocalization of PSD-95 and CaMKIIα before and after induction of chemical long-term potentiation (cLTP), cultures were fixed and immunostained with antibodies targeting MAP2, CaMKIIα, and PSD-95. To minimize spectral crosstalk between fluorescent channels, Alexa Fluor 405, Cy3, and Cy5-conjugated secondary antibodies were employed. Regions of interest (ROIs) within primary dendrites were randomly selected based on MAP2 fluorescence. Pearson’s correlation coefficients for colocalization between CaMKIIα and PSD-95 were calculated using ImageJ software. Data visualization and statistical comparisons were performed using Graphpad Prism 9 (Graphpad Software, Inc., United States).

### Mitochondrial particle analysis in single astrocyte

To assess the functionality of the mitochondrial network in astrocytes, mitochondria were labeled with COX4, an inner mitochondrial membrane protein, and astrocytes were identified using GFAP. Fluorescence images were randomly captured using a ZEISS Apotome 2 microscope equipped with a 20× objective [Plan-Apochromat 20×/0.8 (420650-9902)], at Z-stack 1 μm. For each astrocyte, regions of interest (ROIs) were manually selected based on GFAP staining. The corresponding COX4 channel was then analyzed for mitochondrial particles using ImageJ plugins with a uniform threshold setting.

### Metabolite extraction and sample preparation

Metabolite extraction was performed as previously described (Cordes and Metallo, 2019). Briefly, culture media from two wells of a 24-well plate were pooled into one tube for medium extraction. Each well was quickly washed once with 1 mL of 0.9% NaCl, then quenched with 125 μL of cold methanol and 50 μL of ice-cold water containing 5 μg/mL norvaline (internal standard). Cell extracts from two wells were combined, mixed with 250 μL of cold chloroform (−20 °C), vortexed for 10 min at 4 °C, and centrifuged at 17,000×*g* for 5 min at 4 °C. The upper aqueous phase (200 μL) was collected and evaporated under vacuum in a SpeedVac at 4 °C.

### Gas chromatograph-mass spectrometry (GC–MS) and data analysis

Polar metabolites were derivatized using a Gerstel MPS system. Briefly, 15 μL of 2% (w/v) methoxyamine hydrochloride (Sigma-Aldrich, 226904) in pyridine was added and incubated for 90 min at 55 °C. This was followed by 15 μL of N-tertbutyldimethylsilyl-N-methyltrifluoroacetamide (MTBSTFA) with 1% tert-butyldimethylchlorosilane (TBDMS) (Restek Corporation, 35601) and incubated for 60 min at 55 °C. Derivatized samples were analyzed using an Agilent 7890B gas chromatograph coupled to an Agilent 5977B MSD mass spectrometer with a ZB-35MS column (Phenomenex, 7HG-G003-11-GGA-C). The GC–MS was operated at 70 eV with the MS source temperature set to 230 °C and the quadrupole at 150 °C. Helium served as the carrier gas. The GC oven temperature program was as follows: hold at 100 °C for 2 min, ramp to 180 °C at 10 °C/min, then to 300 °C at 5 °C/min, and finally hold at 325 °C for 3 min.

Metabolite abundances were quantified using in-house algorithms (Cordes and Metallo, 2019) and normalized to the internal standard norvaline, and total protein amount.

### Mendelian randomization (MR) analysis

To explore potential causal relationships between metabolomics-derived amino acid levels and neurodegenerative diseases, we performed Mendelian Randomization (MR) analyses utilizing multiple approaches, including inverse variance weighted (IVW), MR-Egger, weighted median, weighted mode, and simple mode methods. Sensitivity analyses were conducted to evaluate heterogeneity, horizontal pleiotropy, and the overall robustness of the results.

MR analysis relies on three fundamental assumptions. Firstly, the relevance assumption requires that the selected instrumental variables (IVs), specifically single nucleotide polymorphisms (SNPs), are strongly correlated with the exposure of interest—in this case, amino acid levels. Secondly, the independence assumption stipulates that these IVs are not associated with any confounders that could influence both the exposure and the outcome. Lastly, the exclusion restriction assumption posits that the IVs affect the outcome solely through their effect on the exposure, without any direct or alternative pathways (Wagner et al., 2011). Following the STROBE-MR guidelines (Skrivankova et al., 2021), we selected SNPs significantly associated with 18 amino acids at a genome-wide significance threshold (*p* < 5 × 10^−6^) to serve as IVs (Ji et al., 2024; Zhao et al., 2024). These SNPs were further pruned for linkage disequilibrium, retaining those with an *r*^2^ < 0.001 within a 10,000 kb window to ensure the independence of the instrumental variables.

A causal association was deemed statistically significant if the IVW method yielded a *p*-value below 0.05. To enhance the reliability of our causal inferences, additional MR methods (MR-Egger, weighted median, weighted mode, and simple mode) were employed. All analyses were conducted using R software (version 4.2.3) with the TwoSampleMR package (version 0.5.8) and the MRPRESSO package (version 1.0).

### Data presentation and statistical analysis

Data analysis was performed using Graphpad Prism 9 (Graphpad Software, Inc., United States). Data were presented as mean ± SEM. All data were normalized to control for comparison. The dataset first needs to pass a normality test before perfoming either 1-way ANOVA or 2-way ANOVA. For datasets that cannot pass the normality test, corresponding non-normality tests are adopted. The minimum significance value was considered as *p* < 0.05. ns means not significant. All experiments were evaluated in a strictly blind fashion.

### MR analysis

Alzheimer’s Disease (AD):

Protective Effect: Genetic predisposition to lower plasma glycine levels was associated with a reduced risk of AD (OR = 0.951, 95% CI = 0.920–0.983, *p* = 0.003).

Risk Factors: Elevated plasma cysteine (OR = 1.084, 95% CI = 1.009–1.165, *p* = 0.028) and arginine (OR = 1.099, 95% CI = 1.008–1.199, *p* = 0.032) levels increased AD risk.

Amyotrophic Lateral Sclerosis:

Protective Amino Acids: Methionine (OR = 0.870, 95% CI = 0.777–0.975, *p* = 0.017), threonine (OR = 0.935, 95% CI = 0.879–0.995, *p* = 0.034), and leucine (OR = 0.908, 95% CI = 0.829–0.995, *p* = 0.038) demonstrated protective effects against ALS.

Epilepsy and Multiple Sclerosis (MS):

Epilepsy: Higher plasma threonine levels were associated with an increased risk of epilepsy (OR = 1.090, 95% CI = 1.029–1.155, *p* = 0.004).

MS: Elevated serine levels correlated with a higher risk of MS (OR = 1.157, 95% CI = 1.041–1.286, *p* = 0.007).

Parkinson’s Disease (PD):

Risk Factor: Increased plasma threonine levels were also identified as a risk factor for PD (OR = 1.149, 95% CI = 1.013–1.303, *p* = 0.031).

Additional analyses confirmed the robustness of these findings. MR-Egger and MR-PRESSO tests indicated no evidence of horizontal pleiotropy. Cochrane’s Q test revealed no significant heterogeneity (Supplementary Table). Scatter plots illustrated SNP effect sizes for each amino acid across neurodegenerative diseases. Forest plots depicted the causal associations of amino acid-related SNPs, while funnel plots showed no significant heterogeneity among selected instrumental variables. Leave-one-out analysis confirmed that no single SNP biased the estimates.

## Data Availability

The raw data supporting the conclusions of this article will be made available by the authors without undue reservation.
