# Supplementary figures and images for: Ceftriaxone attenuates Poly I:C–induced neuroinflammation in vitro by modulating glutamate transport, synaptic integrity, and immunometabolic reprogramming
Source: Front Cell Neurosci. 2025 Oct 28;19:1684398. doi: 10.3389/fncel.2025.1684398 (PMC12602477; doi:10.3389/fncel.2025.1684398)

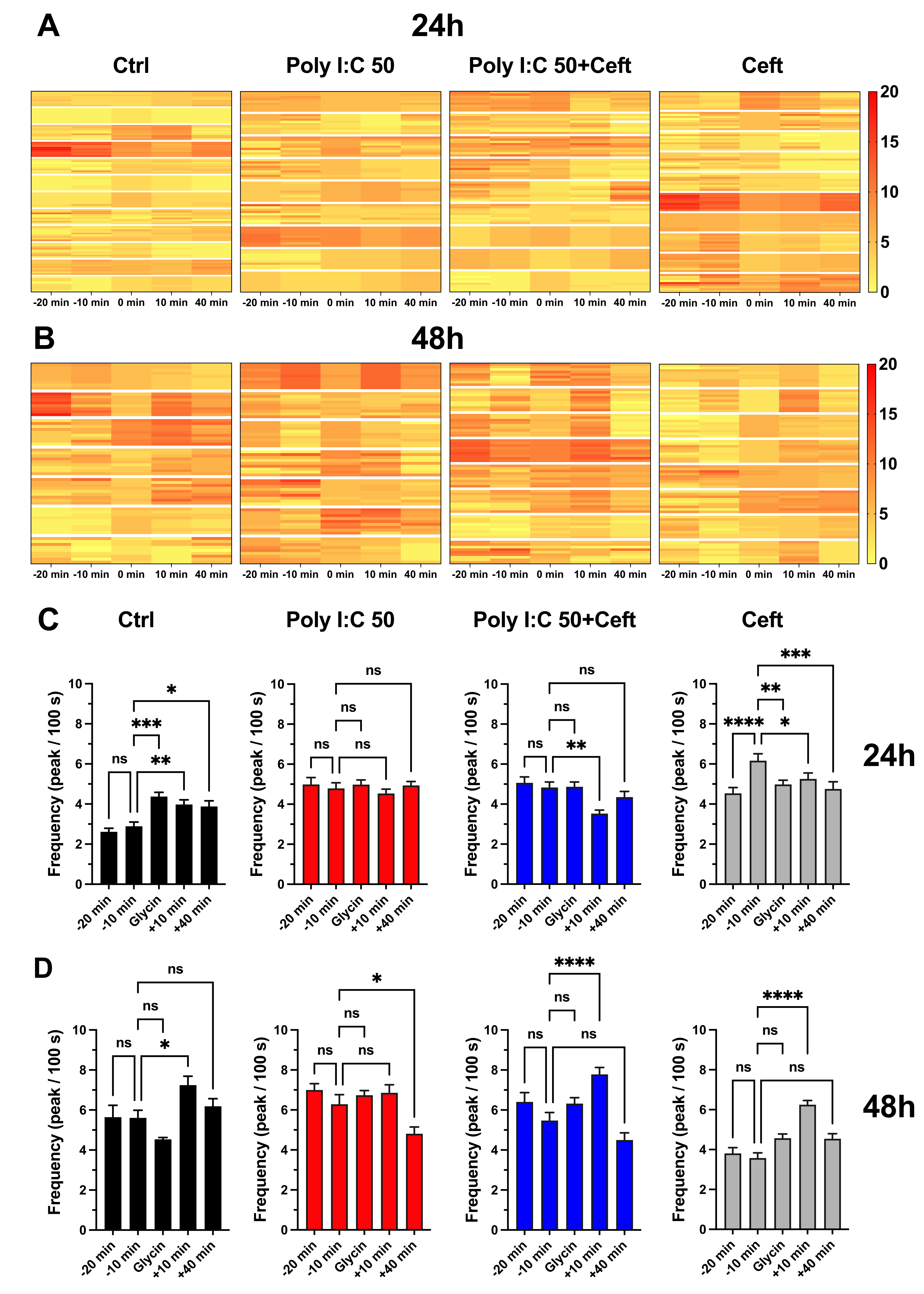

Supplement: Supplementary file 1 [file Image_1.tiff]

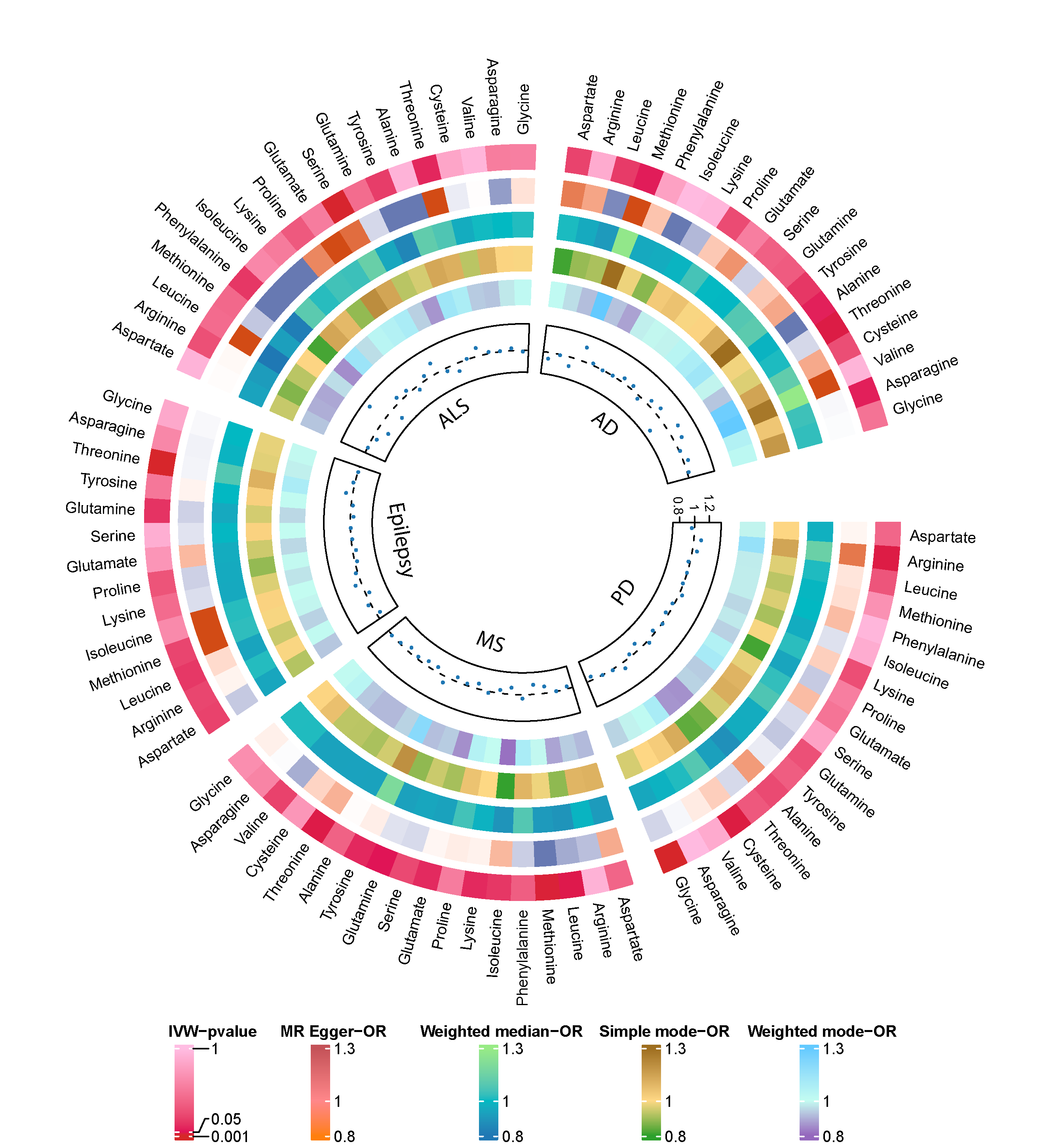

Supplement: Supplementary file 2 [file Image_2.tif]

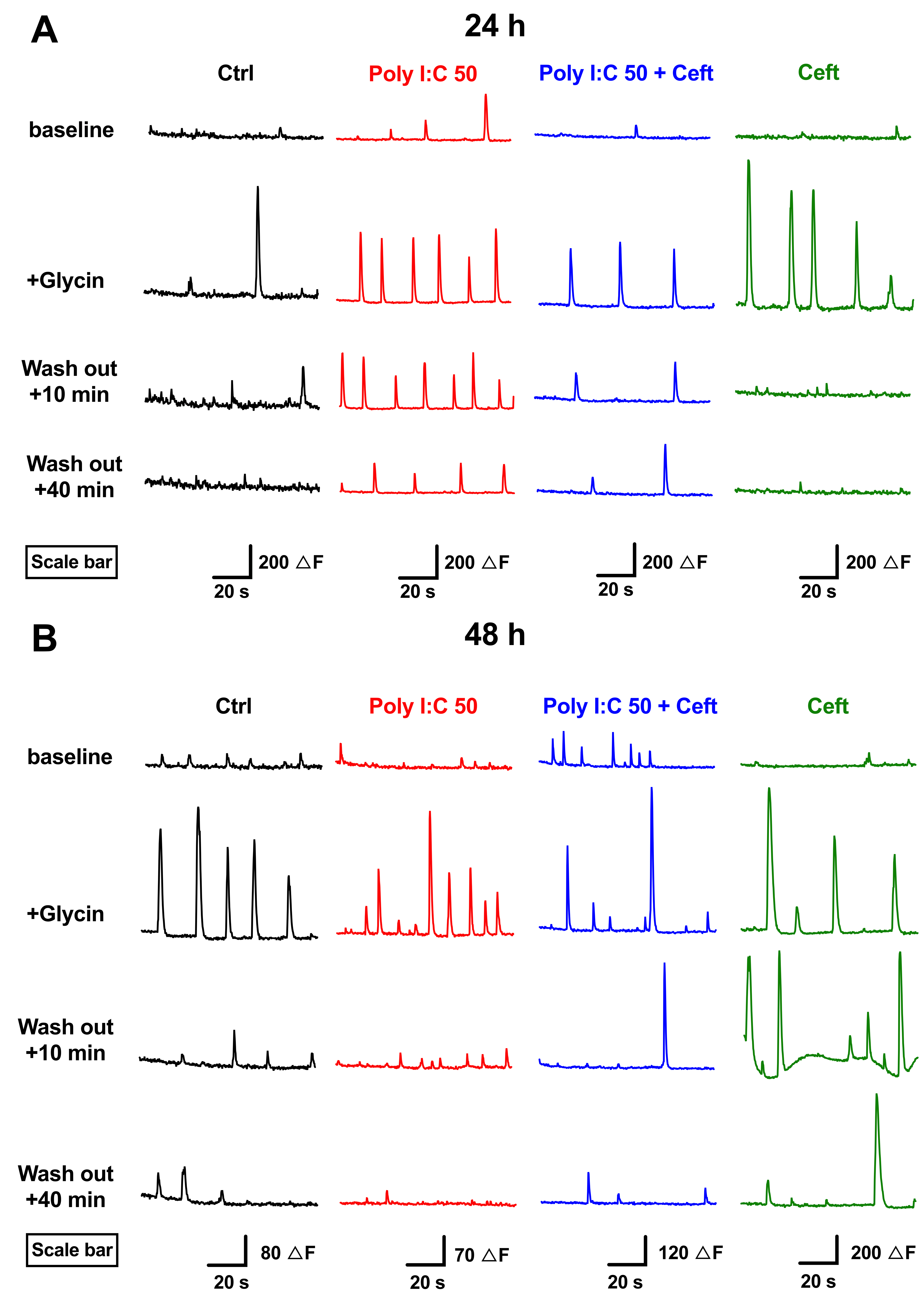

Supplement: Supplementary file 3 [file Image_3.tiff]
